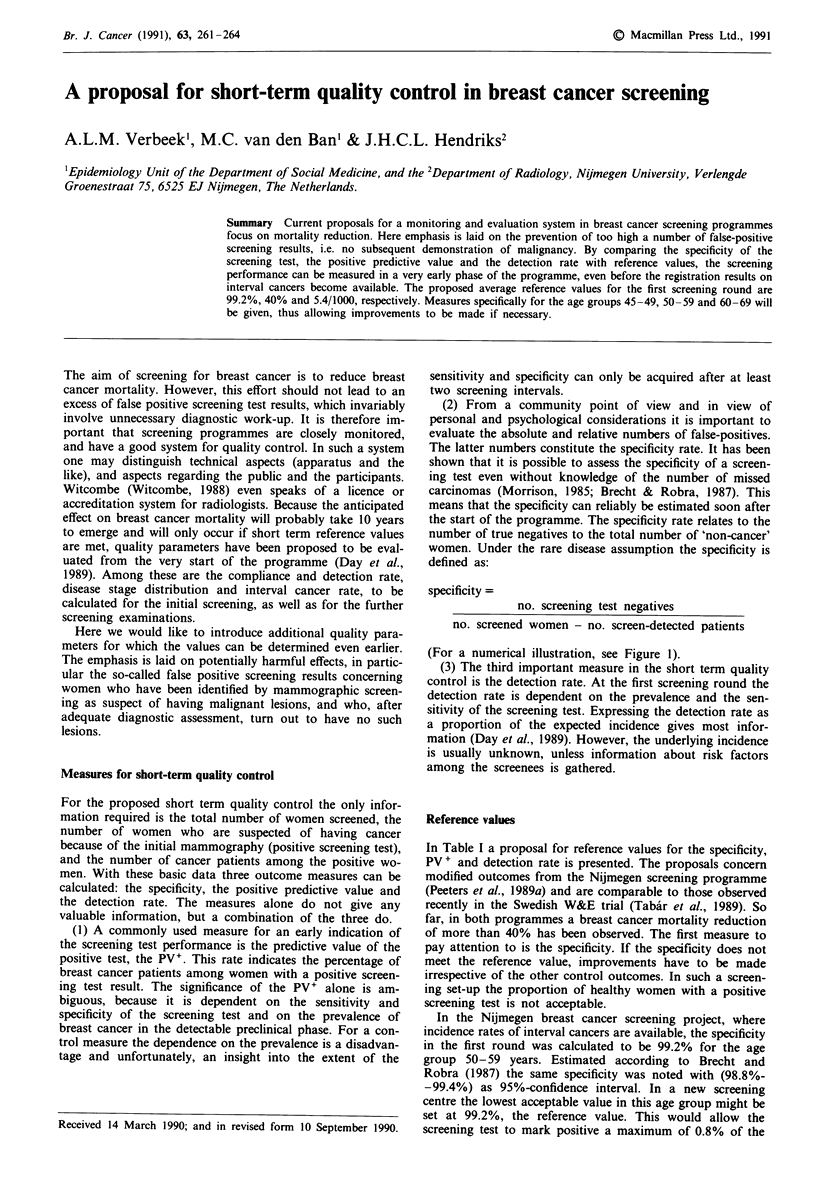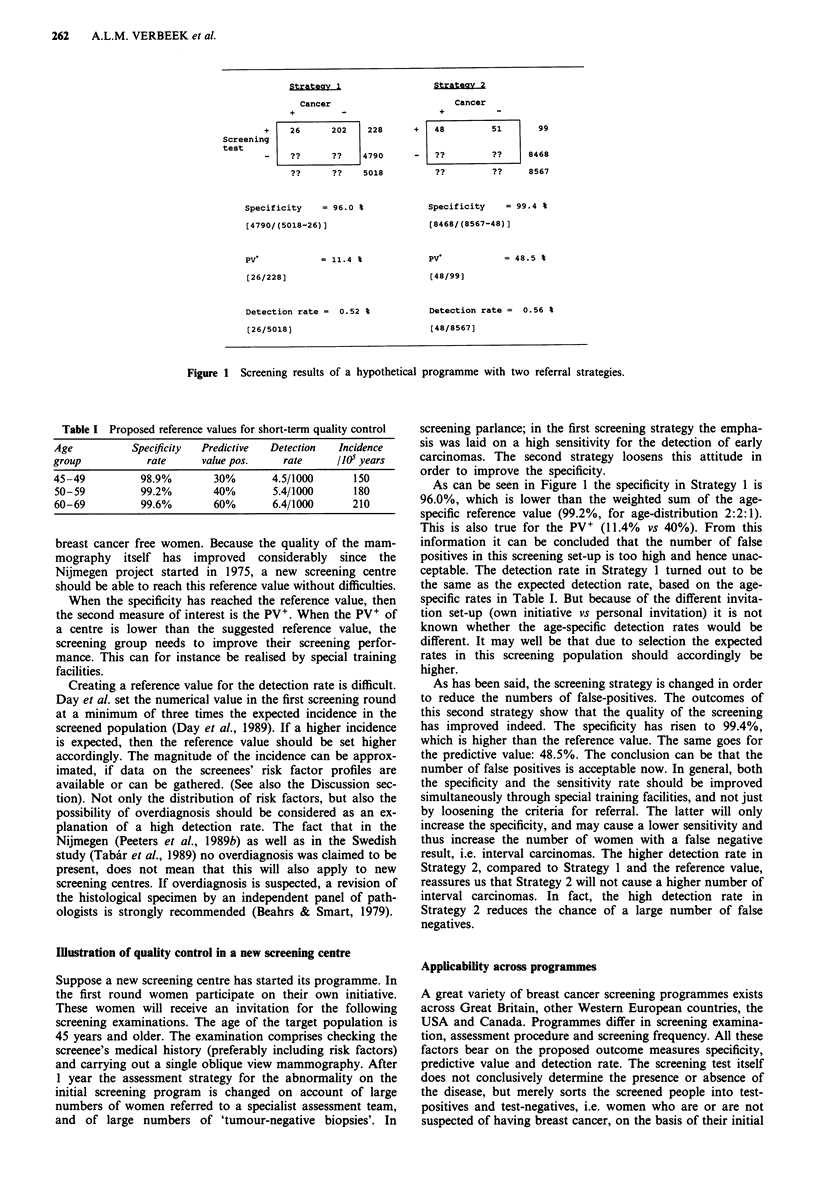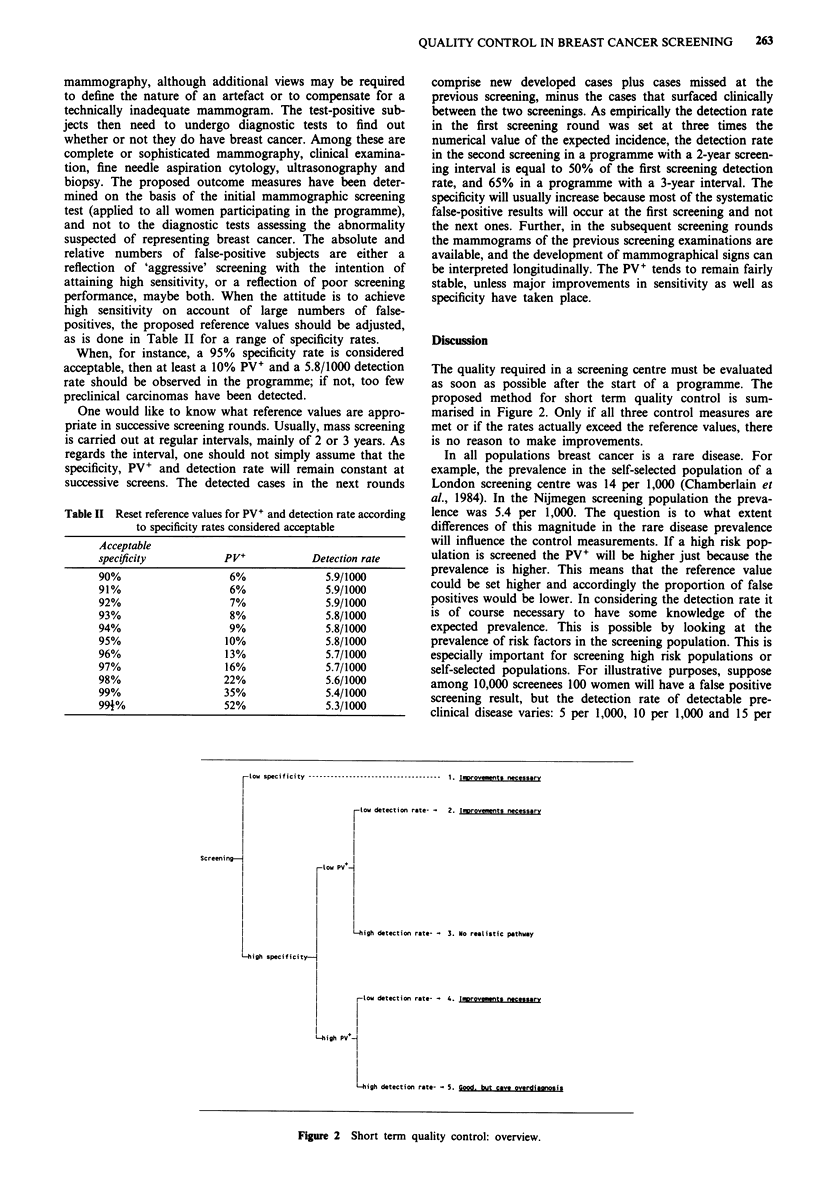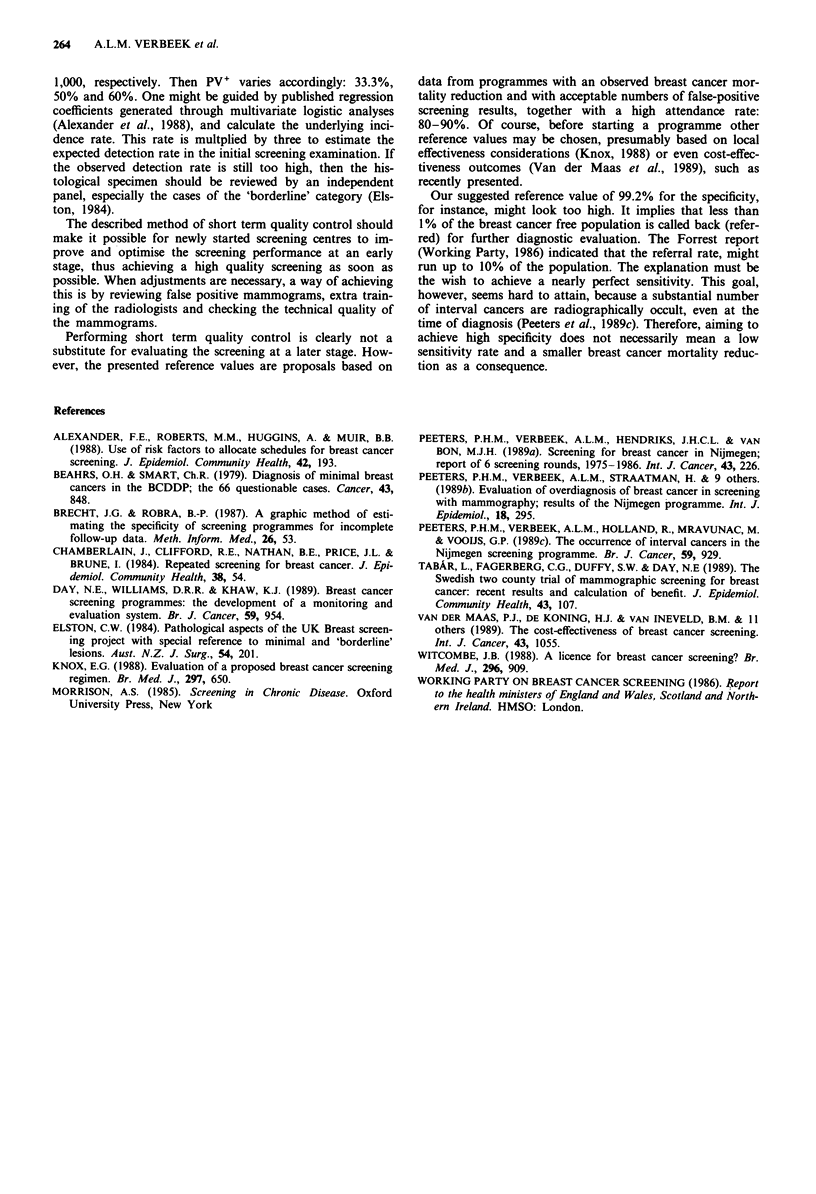# A proposal for short-term quality control in breast cancer screening.

**DOI:** 10.1038/bjc.1991.61

**Published:** 1991-02

**Authors:** A. L. Verbeek, M. C. Van den Ban, J. H. Hendriks

**Affiliations:** Department of Social Medicine, Nijmegen University, The Netherlands.

## Abstract

Current proposals for a monitoring and evaluation system in breast cancer screening programmes focus on mortality reduction. Here emphasis is laid on the prevention of too high a number of false-positive screening results, i.e. no subsequent demonstration of malignancy. By comparing the specificity of the screening test, the positive predictive value and the detection rate with reference values, the screening performance can be measured in a very early phase of the programme, even before the registration results on interval cancers become available. The proposed average reference values for the first screening round are 99.2%, 40% and 5.4/1000, respectively. Measures specifically for the age groups 45-49, 50-59 and 60-69 will be given, thus allowing improvements to be made if necessary.


 © Macmillan Press Ltd., 1991

### A proposal for short-term quality control in breast cancer screening

A.L.M. Verbeek[1], M.C. van den Ban[1] & J.H.C.L. Hendriks[2]

[1]*Epidemiology Unit of the Department of Social Medicine, and the* [2]*Department of Radiology, Nijmegen University, Verlengde Groenestraat 75, 6525 EJ Nijmegen, The Netherlands.*

**Summary** Current proposals for a monitoring and evaluation system in breast cancer screening programmes focus on mortality reduction. Here emphasis is laid on the prevention of too high a number of false-positive screening results, i.e. no subsequent demonstration of malignancy. By comparing the specificity of the screening test, the positive predictive value and the detection rate with reference values, the screening performance can be measured in a very early phase of the programme, even before the registration results on interval cancers become available. The proposed average reference values for the first screening round are 99.2%, 40% and 5.4/1000, respectively. Measures specifically for the age groups 45-49, 50-59 and 60-69 will be given, thus allowing improvements to be made if necessary.

The aim of screening for breast cancer is to reduce breast cancer mortality. However, this effort should not lead to an excess of false positive screening test results, which invariably involve unnecessary diagnostic work-up. It is therefore important that screening programmes are closely monitored, and have a good system for quality control. In such a system one may distinguish technical aspects (apparatus and the like), and aspects regarding the public and the participants. Witcombe (Witcombe, 1988) even speaks of a licence or accreditation system for radiologists. Because the anticipated effect on breast cancer mortality will probably take 10 years to emerge and will only occur if short term reference values are met, quality parameters have been proposed to be evaluated from the very start of the programme (Day *et al.*, 1989). Among these are the compliance and detection rate, disease stage distribution and interval cancer rate, to be calculated for the initial screening, as well as for the further screening examinations.

Here we would like to introduce additional quality parameters for which the values can be determined even earlier. The emphasis is laid on potentially harmful effects, in particular the so-called false positive screening results concerning women who have been identified by mammographic screening as suspect of having malignant lesions, and who, after adequate diagnostic assessment, turn out to have no such lesions.

### Measures for short-term quality control

For the proposed short term quality control the only information required is the total number of women screened, the number of women who are suspected of having cancer because of the initial mammography (positive screening test), and the number of cancer patients among the positive women. With these basic data three outcome measures can be calculated: the specificity, the positive predictive value and the detection rate. The measures alone do not give any valuable information, but a combination of the three do.

(1) A commonly used measure for an early indication of the screening test performance is the predictive value of the positive test, the $PV^+$. This rate indicates the percentage of breast cancer patients among women with a positive screening test result. The significance of the $PV^+$ alone is ambiguous, because it is dependent on the sensitivity and specificity of the screening test and on the prevalence of breast cancer in the detectable preclinical phase. For a control measure the dependence on the prevalence is a disadvantage and unfortunately, an insight into the extent of the sensitivity and specificity can only be acquired after at least two screening intervals.

(2) From a community point of view and in view of personal and psychological considerations it is important to evaluate the absolute and relative numbers of false-positives. The latter numbers constitute the specificity rate. It has been shown that it is possible to assess the specificity of a screening test even without knowledge of the number of missed carcinomas (Morrison, 1985; Brecht & Robra, 1987). This means that the specificity can reliably be estimated soon after the start of the programme. The specificity rate relates to the number of true negatives to the total number of 'non-cancer' women. Under the rare disease assumption the specificity is defined as:

$$\text{specificity} = \frac{\text{no. screening test negatives}}{\text{no. screened women} - \text{no. screen-detected patients}}$$

(For a numerical illustration, see Figure 1).

(3) The third important measure in the short term quality control is the detection rate. At the first screening round the detection rate is dependent on the prevalence and the sensitivity of the screening test. Expressing the detection rate as a proportion of the expected incidence gives most information (Day *et al.*, 1989). However, the underlying incidence is usually unknown, unless information about risk factors among the screenees is gathered.

### Reference values

In Table I a proposal for reference values for the specificity, $PV^+$ and detection rate is presented. The proposals concern modified outcomes from the Nijmegen screening programme (Peeters *et al.*, 1989*a*) and are comparable to those observed recently in the Swedish W&E trial (Tabár *et al.*, 1989). So far, in both programmes a breast cancer mortality reduction of more than 40% has been observed. The first measure to pay attention to is the specificity. If the specificity does not meet the reference value, improvements have to be made irrespective of the other control outcomes. In such a screening set-up the proportion of healthy women with a positive screening test is not acceptable.

In the Nijmegen breast cancer screening project, where incidence rates of interval cancers are available, the specificity in the first round was calculated to be 99.2% for the age group 50-59 years. Estimated according to Brecht and Robra (1987) the same specificity was noted with (98.8%-99.4%) as 95%-confidence interval. In a new screening centre the lowest acceptable value in this age group might be set at 99.2%, the reference value. This would allow the screening test to mark positive a maximum of 0.8% of the

Received 14 March 1990; and in revised form 10 September 1990.

**Strategy 1**

| | | Cancer + | Cancer - | |
|---|---|---|---|---|
| Screening test | + | 26 | 202 | 228 |
| | - | ?? | ?? | 4790 |
| | | ?? | ?? | 5018 |

Specificity = 96.0 %
[4790/(5018-26)]

PV⁺ = 11.4 %
[26/228]

Detection rate = 0.52 %
[26/5018]

**Strategy 2**

| | | Cancer + | Cancer - | |
|---|---|---|---|---|
| Screening test | + | 48 | 51 | 99 |
| | - | ?? | ?? | 8468 |
| | | ?? | ?? | 8567 |

Specificity = 99.4 %
[8468/(8567-48)]

$PV^+$ = 48.5 %
[48/99]

Detection rate = 0.56 %
[48/8567]

**Figure 1** Screening results of a hypothetical programme with two referral strategies.

**Table I** Proposed reference values for short-term quality control

| *Age group* | *Specificity rate* | *Predictive value pos.* | *Detection rate* | *Incidence /10⁵ years* |
|---|---|---|---|---|
| 45–49 | 98.9% | 30% | 4.5/1000 | 150 |
| 50–59 | 99.2% | 40% | 5.4/1000 | 180 |
| 60–69 | 99.6% | 60% | 6.4/1000 | 210 |

breast cancer free women. Because the quality of the mammography itself has improved considerably since the Nijmegen project started in 1975, a new screening centre should be able to reach this reference value without difficulties.

When the specificity has reached the reference value, then the second measure of interest is the $PV^+$. When the $PV^+$ of a centre is lower than the suggested reference value, the screening group needs to improve their screening performance. This can for instance be realised by special training facilities.

Creating a reference value for the detection rate is difficult. Day *et al.* set the numerical value in the first screening round at a minimum of three times the expected incidence in the screened population (Day *et al.*, 1989). If a higher incidence is expected, then the reference value should be set higher accordingly. The magnitude of the incidence can be approximated, if data on the screenees' risk factor profiles are available or can be gathered. (See also the Discussion section). Not only the distribution of risk factors, but also the possibility of overdiagnosis should be considered as an explanation of a high detection rate. The fact that in the Nijmegen (Peeters *et al.*, 1989*b*) as well as in the Swedish study (Tabár *et al.*, 1989) no overdiagnosis was claimed to be present, does not mean that this will also apply to new screening centres. If overdiagnosis is suspected, a revision of the histological specimen by an independent panel of pathologists is strongly recommended (Beahrs & Smart, 1979).

### Illustration of quality control in a new screening centre

Suppose a new screening centre has started its programme. In the first round women participate on their own initiative. These women will receive an invitation for the following screening examinations. The age of the target population is 45 years and older. The examination comprises checking the screenee's medical history (preferably including risk factors) and carrying out a single oblique view mammography. After 1 year the assessment strategy for the abnormality on the initial screening program is changed on account of large numbers of women referred to a specialist assessment team, and of large numbers of 'tumour-negative biopsies'. In screening parlance; in the first screening strategy the emphasis was laid on a high sensitivity for the detection of early carcinomas. The second strategy loosens this attitude in order to improve the specificity.

As can be seen in Figure 1 the specificity in Strategy 1 is 96.0%, which is lower than the weighted sum of the age-specific reference value (99.2%, for age-distribution 2:2:1). This is also true for the $PV^+$ (11.4% *vs* 40%). From this information it can be concluded that the number of false positives in this screening set-up is too high and hence unacceptable. The detection rate in Strategy 1 turned out to be the same as the expected detection rate, based on the age-specific rates in Table I. But because of the different invitation set-up (own initiative *vs* personal invitation) it is not known whether the age-specific detection rates would be different. It may well be that due to selection the expected rates in this screening population should accordingly be higher.

As has been said, the screening strategy is changed in order to reduce the numbers of false-positives. The outcomes of this second strategy show that the quality of the screening has improved indeed. The specificity has risen to 99.4%, which is higher than the reference value. The same goes for the predictive value: 48.5%. The conclusion can be that the number of false positives is acceptable now. In general, both the specificity and the sensitivity rate should be improved simultaneously through special training facilities, and not just by loosening the criteria for referral. The latter will only increase the specificity, and may cause a lower sensitivity and thus increase the number of women with a false negative result, i.e. interval carcinomas. The higher detection rate in Strategy 2, compared to Strategy 1 and the reference value, reassures us that Strategy 2 will not cause a higher number of interval carcinomas. In fact, the high detection rate in Strategy 2 reduces the chance of a large number of false negatives.

### Applicability across programmes

A great variety of breast cancer screening programmes exists across Great Britain, other Western European countries, the USA and Canada. Programmes differ in screening examination, assessment procedure and screening frequency. All these factors bear on the proposed outcome measures specificity, predictive value and detection rate. The screening test itself does not conclusively determine the presence or absence of the disease, but merely sorts the screened people into test-positives and test-negatives, i.e. women who are or are not suspected of having breast cancer, on the basis of their initial mammography, although additional views may be required to define the nature of an artefact or to compensate for a technically inadequate mammogram. The test-positive subjects then need to undergo diagnostic tests to find out whether or not they do have breast cancer. Among these are complete or sophisticated mammography, clinical examination, fine needle aspiration cytology, ultrasonography and biopsy. The proposed outcome measures have been determined on the basis of the initial mammographic screening test (applied to all women participating in the programme), and not to the diagnostic tests assessing the abnormality suspected of representing breast cancer. The absolute and relative numbers of false-positive subjects are either a reflection of 'aggressive' screening with the intention of attaining high sensitivity, or a reflection of poor screening performance, maybe both. When the attitude is to achieve high sensitivity on account of large numbers of false-positives, the proposed reference values should be adjusted, as is done in Table II for a range of specificity rates.

When, for instance, a 95% specificity rate is considered acceptable, then at least a 10% $PV^+$ and a 5.8/1000 detection rate should be observed in the programme; if not, too few preclinical carcinomas have been detected.

One would like to know what reference values are appropriate in successive screening rounds. Usually, mass screening is carried out at regular intervals, mainly of 2 or 3 years. As regards the interval, one should not simply assume that the specificity, $PV^+$ and detection rate will remain constant at successive screens. The detected cases in the next rounds comprise new developed cases plus cases missed at the previous screening, minus the cases that surfaced clinically between the two screenings. As empirically the detection rate in the first screening round was set at three times the numerical value of the expected incidence, the detection rate in the second screening in a programme with a 2-year screening interval is equal to 50% of the first screening detection rate, and 65% in a programme with a 3-year interval. The specificity will usually increase because most of the systematic false-positive results will occur at the first screening and not the next ones. Further, in the subsequent screening rounds the mammograms of the previous screening examinations are available, and the development of mammographical signs can be interpreted longitudinally. The $PV^+$ tends to remain fairly stable, unless major improvements in sensitivity as well as specificity have taken place.

**Table II** Reset reference values for $PV^+$ and detection rate according to specificity rates considered acceptable

| *Acceptable specificity* | $PV^+$ | *Detection rate* |
|---|---|---|
| 90% | 6% | 5.9/1000 |
| 91% | 6% | 5.9/1000 |
| 92% | 7% | 5.9/1000 |
| 93% | 8% | 5.8/1000 |
| 94% | 9% | 5.8/1000 |
| 95% | 10% | 5.8/1000 |
| 96% | 13% | 5.7/1000 |
| 97% | 16% | 5.7/1000 |
| 98% | 22% | 5.6/1000 |
| 99% | 35% | 5.4/1000 |
| 99½% | 52% | 5.3/1000 |

### Discussion

The quality required in a screening centre must be evaluated as soon as possible after the start of a programme. The proposed method for short term quality control is summarised in Figure 2. Only if all three control measures are met or if the rates actually exceed the reference values, there is no reason to make improvements.

In all populations breast cancer is a rare disease. For example, the prevalence in the self-selected population of a London screening centre was 14 per 1,000 (Chamberlain *et al.*, 1984). In the Nijmegen screening population the prevalence was 5.4 per 1,000. The question is to what extent differences of this magnitude in the rare disease prevalence will influence the control measurements. If a high risk population is screened the $PV^+$ will be higher just because the prevalence is higher. This means that the reference value could be set higher and accordingly the proportion of false positives would be lower. In considering the detection rate it is of course necessary to have some knowledge of the expected prevalence. This is possible by looking at the prevalence of risk factors in the screening population. This is especially important for screening high risk populations or self-selected populations. For illustrative purposes, suppose among 10,000 screenees 100 women will have a false positive screening result, but the detection rate of detectable preclinical disease varies: 5 per 1,000, 10 per 1,000 and 15 per

**Figure 2** Short term quality control: overview.

1,000, respectively. Then $PV^+$ varies accordingly: 33.3%, 50% and 60%. One might be guided by published regression coefficients generated through multivariate logistic analyses (Alexander *et al.*, 1988), and calculate the underlying incidence rate. This rate is multplied by three to estimate the expected detection rate in the initial screening examination. If the observed detection rate is still too high, then the histological specimen should be reviewed by an independent panel, especially the cases of the 'borderline' category (Elston, 1984).

The described method of short term quality control should make it possible for newly started screening centres to improve and optimise the screening performance at an early stage, thus achieving a high quality screening as soon as possible. When adjustments are necessary, a way of achieving this is by reviewing false positive mammograms, extra training of the radiologists and checking the technical quality of the mammograms.

Performing short term quality control is clearly not a substitute for evaluating the screening at a later stage. However, the presented reference values are proposals based on data from programmes with an observed breast cancer mortality reduction and with acceptable numbers of false-positive screening results, together with a high attendance rate: 80–90%. Of course, before starting a programme other reference values may be chosen, presumably based on local effectiveness considerations (Knox, 1988) or even cost-effectiveness outcomes (Van der Maas *et al.*, 1989), such as recently presented.

Our suggested reference value of 99.2% for the specificity, for instance, might look too high. It implies that less than 1% of the breast cancer free population is called back (referred) for further diagnostic evaluation. The Forrest report (Working Party, 1986) indicated that the referral rate, might run up to 10% of the population. The explanation must be the wish to achieve a nearly perfect sensitivity. This goal, however, seems hard to attain, because a substantial number of interval cancers are radiographically occult, even at the time of diagnosis (Peeters *et al.*, 1989*c*). Therefore, aiming to achieve high specificity does not necessarily mean a low sensitivity rate and a smaller breast cancer mortality reduction as a consequence.

### References

ALEXANDER, F.E., ROBERTS, M.M., HUGGINS, A. & MUIR, B.B. (1988). Use of risk factors to allocate schedules for breast cancer screening. *J. Epidemiol. Community Health*, **42**, 193.

BEAHRS, O.H. & SMART, Ch.R. (1979). Diagnosis of minimal breast cancers in the BCDDP; the 66 questionable cases. *Cancer*, **43**, 848.

BRECHT, J.G. & ROBRA, B.-P. (1987). A graphic method of estimating the specificity of screening programmes for incomplete follow-up data. *Meth. Inform. Med.*, **26**, 53.

CHAMBERLAIN, J., CLIFFORD, R.E., NATHAN, B.E., PRICE, J.L. & BRUNE, I. (1984). Repeated screening for breast cancer. *J. Epidemiol. Community Health*, **38**, 54.

DAY, N.E., WILLIAMS, D.R.R. & KHAW, K.J. (1989). Breast cancer screening programmes: the development of a monitoring and evaluation system. *Br. J. Cancer*, **59**, 954.

ELSTON, C.W. (1984). Pathological aspects of the UK Breast screening project with special reference to minimal and 'borderline' lesions. *Aust. N.Z. J. Surg.*, **54**, 201.

KNOX, E.G. (1988). Evaluation of a proposed breast cancer screening regimen. *Br. Med. J.*, **297**, 650.

MORRISON, A.S. (1985). *Screening in Chronic Disease*. Oxford University Press, New York

PEETERS, P.H.M., VERBEEK, A.L.M., HENDRIKS, J.H.C.L. & VAN BON, M.J.H. (1989*a*). Screening for breast cancer in Nijmegen; report of 6 screening rounds, 1975–1986. *Int. J. Cancer*, **43**, 226.

PEETERS, P.H.M., VERBEEK, A.L.M., STRAATMAN, H. & 9 others. (1989*b*). Evaluation of overdiagnosis of breast cancer in screening with mammography; results of the Nijmegen programme. *Int. J. Epidemiol.*, **18**, 295.

PEETERS, P.H.M., VERBEEK, A.L.M., HOLLAND, R., MRAVUNAC, M. & VOOIJS, G.P. (1989*c*). The occurrence of interval cancers in the Nijmegen screening programme. *Br. J. Cancer*, **59**, 929.

TABÁR, L., FAGERBERG, C.G., DUFFY, S.W. & DAY, N.E (1989). The Swedish two county trial of mammographic screening for breast cancer: recent results and calculation of benefit. *J. Epidemiol. Community Health*, **43**, 107.

VAN DER MAAS, P.J., DE KONING, H.J. & VAN INEVELD, B.M. & 11 others (1989). The cost-effectiveness of breast cancer screening. *Int. J. Cancer*, **43**, 1055.

WITCOMBE, J.B. (1988). A licence for breast cancer screening? *Br. Med. J.*, **296**, 909.

WORKING PARTY ON BREAST CANCER SCREENING (1986). *Report to the health ministers of England and Wales, Scotland and Northern Ireland*. HMSO: London.